# Combining Coagulation/MIEX with Biological Activated Carbon Treatment to Control Organic Fouling in the Microfiltration of Secondary Effluent

**DOI:** 10.3390/membranes6030039

**Published:** 2016-07-30

**Authors:** Biplob Kumar Pramanik, Felicity A. Roddick, Linhua Fan

**Affiliations:** School of Engineering, RMIT University, GPO Box 2476, Melbourne 3001, Australia; s3354492@student.rmit.edu.au (B.K.P.); linhua.fan@rmit.edu.au (L.F.)

**Keywords:** biological activated carbon, biopolymers, coagulation, magnetic ion exchange, membrane fouling, secondary effluent

## Abstract

Coagulation, magnetic ion exchange resin (MIEX) and biological activated carbon (BAC) were examined at lab scale as standalone, and sequential pre-treatments for controlling the organic fouling of a microfiltration membrane by biologically treated secondary effluent (BTSE) using a multi-cycle approach. MIEX gave slightly greater enhancement in flux than coagulation due to greater removal of high molecular weight (MW) humic substances, although it was unable to remove high MW biopolymers. BAC treatment was considerably more effective for improving the flux than coagulation or MIEX. This was due to the biodegradation of biopolymers and/or their adsorption by the biofilm, and adsorption of humic substances by the activated carbon, as indicated by size exclusion chromatography. Coagulation or MIEX followed by BAC treatment further reduced the problematic foulants and significantly improved the flux performance. The unified membrane fouling index showed that the reduction of membrane fouling by standalone BAC treatment was 42%. This improved to 65%, 70%, and 93% for alum, ferric chloride and MIEX pre-treatment, respectively, when followed by BAC treatment. This study showed the potential of sequential MIEX and BAC pre-treatment for controlling organic fouling and thus enhancing the performance of microfiltration in the reclamation of BTSE.

## 1. Introduction

Low pressure membrane (LPM) processes such as microfiltration (MF) and ultrafiltration (UF) systems are attractive and reliable for the treatment of potable water and secondary effluent since they have many advantages such as small footprint, high pollutant removal efficiency, low energy consumption and good mechanical and chemical stability. However, these membranes are subject to fouling by effluent organic matter (EfOM) during the filtration of biologically treated secondary effluent (BTSE) [1,2], since it contains nucleic acids, polysaccharides, proteins, amino-sugars, humic materials, cell components and organic acids [3]. This membrane fouling is a major barrier to the application of these processes, as it can reduce permeability and so increase the need for frequent hydraulic backwashing and/or chemical cleaning which leads to increased operational costs and reduced membrane life span. Therefore an appropriate pre-treatment is required for the successful application of LPM systems.

Pre-treatment of feedwater is widely used to reduce the organic load on the membrane and to modify the character of the organics [4,5], and thus to reduce membrane fouling. Coagulation has been used to mitigate membrane fouling during drinking water and secondary effluent treatment. Fan et al. [6] found that coagulation with either alum or ferric chloride could improve the flux performance for MF and UF of BTSE. This was due to the removal of major foulants such as biopolymers and humic substances. The efficacy of organics removal by coagulation varies according to the characteristics of the feedwater, and the dose and type of the coagulant [7]. Some studies found that in-line coagulation (i.e., low dosages) had a negative impact on membrane performance [8,9] since it led to the aggregation of particles into larger masses and hence greater filter cake resistance. However, different pre-treatments have different capacities for the removal of the various types of organics and so have a different effect on membrane fouling. There has been limited investigation of the removal of different fractions of organic matter from BTSE by coagulation and the subsequent impact on LPM fouling.

Anion exchange resin (AER) is widely used as a simple and effective means for the removal of organic materials from water and wastewater. Filloux et al. [10] reported that conventional AER did not improve the flux performance for MF/UF of secondary effluent. However, Myat et al. [11] found that magnetic anion exchange resin (MIEX) could improve both UF and MF performance for secondary effluent treatment. Kabsch-Korbutowicz et al. [12] noted that the MIEX process is better for removing organics than conventional AER as it has 2–5 times greater external surface area and thus allows faster sorption kinetics. Moreover, it contains a high proportion of magnetic iron oxide, which can transform the agglomerate of fine resin beads into larger, fast-settling particles. Aryal et al. [13] found that biological activated carbon (BAC) and MIEX pre-treatment (individually and in combination) of secondary effluent was effective for controlling the fouling of a nanofiltration membrane. However, only a few studies have been conducted on MIEX treatment for mitigating LPM membrane fouling.

A study by Marco et al. [14] suggested that the operating and capital costs of physico-chemical processes are markedly higher (3–10 and 5–20 times, respectively) than for biological treatment processes such as slow sand filtration (SSF) and BAC. BAC appears to be a better solution for foulant mitigation compared with SSF as BAC can lead to both physico-chemical adsorption of humic substances and biodegradation of the biopolymers, whereas SSF was found to be ineffective for the removal of non-biodegradable humic substances [15]. It has been demonstrated that BAC treatment was very effective for degrading high MW biopolymers and adsorbing humic substances from a BTSE [16,17], thereby contributing to improvement in the flux. Pramanik et al. [18] also noted that the flux improvement decreased with the service time of the BAC column, which was due to decreased removal of humic substances as a result of gradual reduction in the adsorption capacity of the activated carbon. Therefore, coagulation or MIEX followed by BAC filtration could be used to remove the humics and so enable sustainable application of MF processes to secondary effluent, since both coagulation and MIEX preferentially remove high MW humic substances.

The objective of the present work was to compare the effect of coagulation, MIEX and BAC as feedwater pre-treatments for improving microfiltration performance for the treatment of a BTSE. The effect of sequential coagulation-BAC and MIEX-BAC treatments was also investigated to determine the benefits of the treatment combinations in removing different types of organic foulants.

## 2. Experimental Section

### 2.1. BTSE Source

The BTSE was taken from a local wastewater treatment plant that utilizes an activated sludge-lagoon treatment process. After passing through activated sludge ponds with anoxic and aerobic zones, the effluent enters a clarifier. The clarified stream then passes through a series of lagoons and is collected in storage ponds, and is disinfected on release for reuse. The sample was kept at 4 °C and warmed to room temperature (22 ± 2 °C) prior to all experiments. The pH of the BTSE was 7.6.

### 2.2. Pre-Treatment

#### 2.2.1. BAC Treatment

The BAC column was glass, with an internal diameter of 2.3 cm and carbon bed height of 22 cm. The column was run in down flow mode, and empty bed contact time was 40 min. The column was backwashed every 14 days for 10 min to prevent clogging.

A coal-based granular activated carbon (GAC 1300, Activated Carbon Technology, Melbourne, Australia) was utilized, and its characteristics are shown in Table S1 (Properties of the activated carbon used in the column). Before packing the column, the GAC was inoculated with activated sludge which had been supplemented with nutrients (C, P and N as described by Lu et al. [19]) and aerated for five days to promote biofilm growth on the carbon surface. It was then gently washed using Milli-Q (Millipore, Darmstadt, Germany) water to reduce excess biofilm and transferred to the BAC column, and BTSE feed commenced. After 90 days, the reduction in dissolved organic carbon (DOC) was consistent (30% ± 3%), indicating that stable function of the system had been established. The results reported here are for samples collected after the column had run continuously for 840 days.

#### 2.2.2. Coagulation

Coagulation was performed in a jar test apparatus (Phipps and Bird, PB-700, Richmond, VA, USA) at room temperature (20 ± 2 °C). The coagulant was alum (Al_2_(SO_4_)_3_·18H_2_O) or ferric chloride (FeCl_3_·6H_2_O) (Chem-Supply, Pty Ltd., Adelaide, Australia). The samples were mixed for 2 min at 250 rpm, slow mixed for 30 min at 30 rpm, and then allowed to settle for 2 h before taking the supernatant for experiments. This experiment was performed at pH 5 with a dose of 5 mg/L (Al^3+^ or Fe^3+^) based on chemical used and DOC removal (Table S2 DOC removal using alum and ferric chloride coagulation). Prior to use, the supernatant was adjusted to pH 7.6 with 1 M NaOH to make it the same as the un-treated BTSE.

#### 2.2.3. MIEX Treatment

MIEX resin was supplied by IXOM, Melbourne, Australia. Prior to the tests, the resin was thoroughly washed with Milli-Q water. A range of dosages was tested (2–12 mL/L) and 10 mL/L was used as maximum DOC removal occurred at this dose. Resin was added to 2 L of BTSE in the laboratory jar test apparatus and the samples were mixed for 30 min at 170 rpm as recommended by IXOM, and then settled for 5 min before taking supernatant samples. Prior to the BAC or filtration tests, resin fines were removed by filtration (1 μm, Whatman GF/C, Sigma-Aldrich, St. Louis, MO, USA).

#### 2.2.4. Microfiltration Experiment Set-Up

Microfiltration experiments were conducted with a stirred cell of dead-end configuration (effective membrane area 13.4 cm^2^, Amicon 8050). A 0.1 μm hydrophilic polyvinylidene fluoride membrane (Millipore, Billerica, MA, USA) was used. The trans-membrane pressure of 50 kPa was maintained using compressed nitrogen gas, and the stirring speed was 430 rpm. A digital balance (BS210S, Sartorius, Germany) linked to a computer was used to continuously record permeate weight and thus to monitor the cumulative volumetric flux. These data were used to compute the permeate flux (*J*). Filtration was performed at room temperature (22 ± 2 °C).

Before filtration, membranes were soaked in Milli-Q water for 2 h to remove membrane preservatives, after which 500 mL of Milli-Q water was passed through them to determine the pure water flux (*J_o_*), which varied by only 3%. All MF experiments comprised three consecutive filtration cycles. After each cycle, and after inverting the fouled membrane in the cell, it was backwashed with 50 mL Milli-Q water. On returning it to its original orientation, 100 mL Milli-Q water was then passed through it to enable the determination of the reversibility of fouling. The backwash and permeate samples were analysed to quantify and identify the organics responsible for reversible and irreversible fouling. Trans-membrane pressure was 50 kPa during the backwash procedure. Duplicate filtration runs were conducted for each sample, and as the trends were consistent (final flux varied by ≤4%), only one set of flux data is reported in this paper.

#### 2.2.5. Calculation of Fouling Resistance

The following equation [20] was used to determine both reversible and irreversible fouling resistance:

(1) $$R_{f}=\frac{\Delta P}{µJ}-R_{m}$$

where *R_f_* = fouling resistance (/m); μ = water viscosity (Ns/m^2^, Pa·s) = 497 × 10^3^/(T + 42.5)^1.5^; T = feedwater temperature (°C); ΔP = trans-membrane pressure, TMP (N/m^2^, Pa); *R_m_* = membrane resistance (/m) as calculated from the pure water flux and *J* = permeate flux at the end of filtration run (m^3^/m^2^·s).

### 2.3. Analytical Methods

DOC was measured with a Sievers 820 TOC analyser (Boulder, CO, USA). Absorbance at 254 nm (UVA_254_) was determined with a UV-Vis spectrophotometer (UV2, Unicam) and colour with a Hach spectrophotometer (Model DR/5000) (Ames, IA, USA). Fluorescence excitation-emission matrix (EEM) spectra were obtained with a PerkinElmer Spectrometer (LS55, PerkinElmer, Waltham, MA, USA) over an excitation range of 220–465 nm and emission range of 280–550 nm. All samples were filtered (0.45 μm) prior to these analyses. The pH was determined using a pH meter (Hach Sension, 156, Ames, IA, USA).

The protein concentration in the water samples was determined using the bicinchoninic acid (BCA) method with a QPBCA QuantiPro™ BCA Assay Kit (Sigma Aldrich, St. Louis, MO, USA) and bovine serum albumin (Sigma Aldrich) as the standard protein. The concentration of carbohydrate was measured using the phenol-sulfuric method [21] and d-glucose as the standard carbohydrate.

The apparent molecular weight distribution of the organic content of the water samples was determined by liquid chromatography with organic carbon detection (LC-OCD) (Model 8, DOC-Labor) at the Water Research Centre at the University of New South Wales, Australia. The detailed technique can be found in Huber et al. [22].

Determination of the hydrophobic (HPO), transphilic (TPI) and hydrophilic (HPI) contents of the EfOM was undertaken by use of the non-ionic macroporous resins Amberlite XAD-4 (Supelco, Sigma Aldrich, NSW, Australia) and Supelite DAX-8 (Supelco, Sigma Aldrich, NSW, Australia). The samples were pre-filtered (0.45 μm), adjusted to pH 2 with 2M HCl, and then passed through the DAX-8 followed by the XAD-4 resin at 3 mL/min. The detailed fractionation procedure can be found in Lee et al. [23].

The fouling on the membrane surfaces was analysed using ATR-FTIR (100 FTIR spectrometer, PerkinElmer, Waltham, MA, USA). The membranes were dried for 24 h at room temperature prior to analysis.

## 3. Results and Discussion

### 3.1. Water Quality

The characteristics of the BTSE before and after the different treatments are presented in Table 1. MIEX led to greater removal of DOC, UVA_254_ and colour than BAC treatment, which in turn was greater than for coagulation by alum or ferric chloride. The organic matter removal by MIEX was due to the adsorption of the organics by the resin as well as ion exchange of the negatively charged organic molecules with the cationic functional groups on the resin [12]. The removal by BAC was attributed to the adsorption of some organics by the activated carbon and the biofilm, and the biodegradation of others. Removal of the organics by coagulation involves charge neutralization and the adsorption of organics on the metal hydroxide [24,25]. Coagulation with ferric chloride gave marginally greater removal of DOC, UVA_254_ and colour content compared with alum, which was attributed to the larger floc sizes [25] and higher charge density [26] than for alum. MIEX gave greater reduction in specific ultraviolet absorbance (SUVA) than treatment by either BAC or coagulation, indicating the preferential removal of UV-absorbing organics. Similar findings were noted by Mergen et al. [27] who found that MIEX was more effective than coagulation for the removal of UV-absorbing organic matter from drinking water.

MIEX led to slightly lower removal of protein and carbohydrate content than BAC treatment. This was attributed to these molecules lacking negatively charged functional groups at neutral pH [28]. The removal efficiency for both protein and carbohydrate content was greater for MIEX than for coagulation. Compared with alum, ferric chloride removed more protein and less carbohydrate. BAC gave greater reduction of protein (37%) than carbohydrate content (32%) due to extracellular proteins being more biodegradable than polysaccharide-like materials, as found by Flemming and Wingender [29] and cited by Haberkamp et al. [30].

The sequence of coagulation or MIEX followed by BAC led to further removal of organic compounds. There was greater removal of DOC when alum rather than ferric chloride coagulation preceded BAC, although standalone ferric coagulation gave greater removal of DOC than alum. This was probably because ferric chloride preferentially removes protein whereas alum preferentially removes carbohydrate, so that when the alum-treated sample was passed through the BAC column, there was greater utilization and thus removal of the residual organics than in the ferric chloride-treated sample.

In order to gain further insight into the composition of the organics after the different pre-treatments, the relative proportions of the carbohydrate and protein contents were determined to understand their importance in membrane fouling. After coagulation, the ratios of carbohydrate/DOC and protein/DOC were 0.99 and 1.16 for alum, and 1.09 and 1.13 for ferric chloride, respectively, indicating that the alum-treated effluent had lower proportions of carbohydrate and slightly higher proportions of protein than ferric chloride-treated effluent. For MIEX, the carbohydrate/DOC and protein/DOC ratios were 1.24 and 1.33, respectively, showing higher proportions of both carbohydrate and protein remaining than after coagulation. After BAC treatment, the ratios of carbohydrate/DOC and protein/DOC were 0.96 and 1.06, respectively. Therefore, the ratios of carbohydrate and protein to DOC were lower after BAC than after coagulation, which, in turn, were lower than after MIEX treatment. Sequential treatment gave lower ratios of protein and carbohydrate to DOC than the standalone processes, and MIEX and coagulation followed by BAC gave similar ratios of protein to DOC, whereas MIEX followed by BAC gave a higher ratio of carbohydrate to DOC than coagulation followed by BAC.

### 3.2. Microfiltration Performance after Different Pre-Treatments

#### 3.2.1. Flux Performance

**Figure 1:**
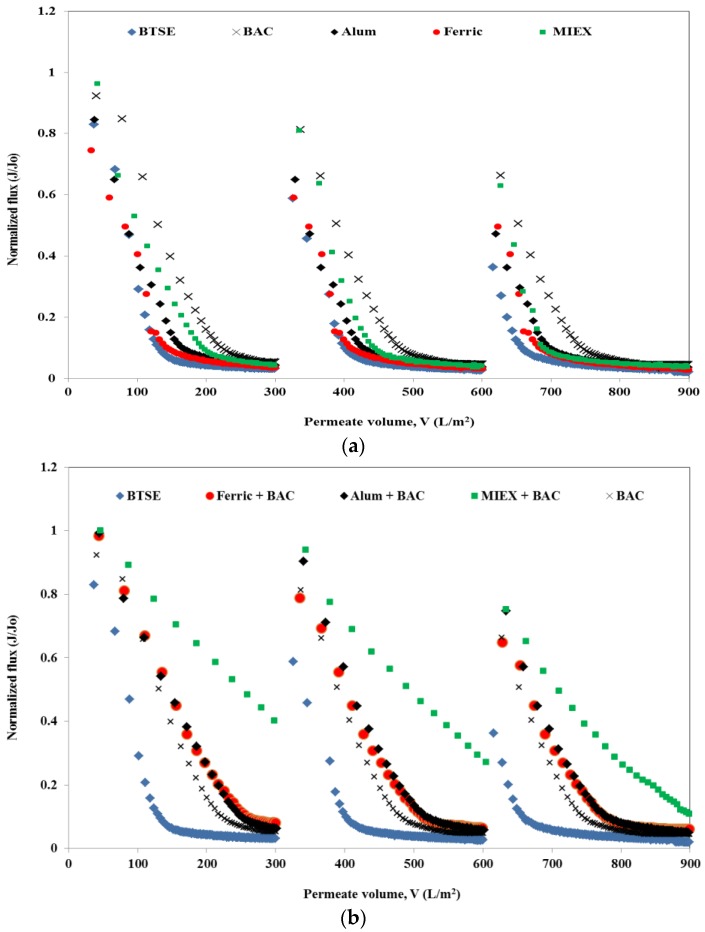
Effect of pre-treatment on flux performance (**a**) standalone treatment and (**b**) combination of different treatments with BAC.

The normalised flux profiles obtained after the various pre-treatments of BTSE are shown in Figure 1. For each cycle, the permeate volume of the MF membrane for both un-treated and treated samples was set at 300 mL. The un-treated BTSE led to severe flux decline, with approximately 95% decrease in flux at the end of the filtration cycle. Feedwater pre-treatment significantly reduced the flux decline, indicating the foulant causing severe flux reduction was removed by the treatment process. Greater flux enhancement was obtained with BAC than with MIEX, which, in turn, was greater than for both coagulants, and for cycle 1 300 mL permeate was achieved after 41 min (Figure 1a). Both coagulated samples had a similar flux decline pattern in the initial 10 min of filtration, however, after this alum gave greater flux improvement than ferric chloride.

Although each standalone pre-treatment led to the removal of some of the organics in the BTSE, the residual organic matter still caused membrane fouling. Therefore, coagulation or MIEX followed by BAC were investigated for the further removal of organics and thus membrane foulants prior to MF. There was a greater improvement in flux after the sequential treatments than the standalone processes. MIEX followed by BAC was more effective than coagulation followed by BAC (Figure 1b). This was primarily due to greater removal of organic content (in terms of DOC, UVA_254_, protein and carbohydrate) (Table 1). Although standalone alum coagulation was better than ferric chloride coagulation for improving the flux, ferric chloride coagulation followed by BAC gave a greater improvement in flux than the alum-BAC process. With successive filtration cycles, there was a greater decline in flux for all feed types. This was due to greater retention of the organics (in terms of DOC, protein and carbohydrate) with successive filtration cycles as shown by mass balances (Figure S1 Distribution of organics in reversible and irreversible fouling after MF). The trend for flux decline for each sample with successive filtration cycles was the same as for cycle 1.

#### 3.2.2. Fouling Resistance

**Figure 2:**
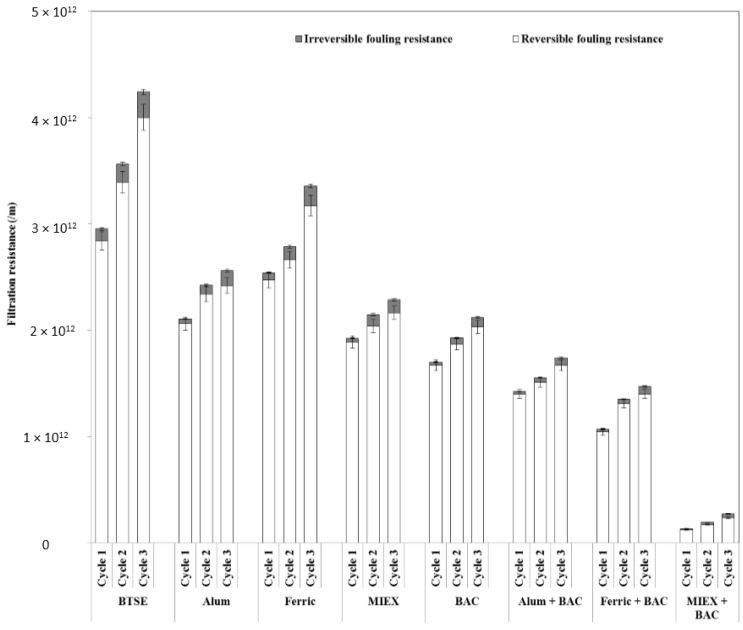
Effect of pre-treatment on fouling reversibility (data points are average values of duplicate samples).

Both reversible and irreversible resistance were decreased markedly after pre-treatment of the feedwater (Figure 2). The reduction after BAC was greater than after the MIEX or coagulation processes. BAC led to a larger reduction in irreversible (73%) than reversible fouling resistance (41%), indicating a decrease in pore blocking and/or pore adsorption of the membranes. The reduction in hydraulically reversible fouling was greater after MIEX than alum treatment, which, in turn, was greater than that after ferric chloride coagulation. Although MIEX and alum gave similar performance in reducing the irreversible fouling, MIEX led to a greater improvement in flux. This was likely due to greater reduction in reversible fouling resistance as it contributed more than 90% of the total filtration resistance. The sequence of MIEX or coagulation followed by BAC treatment gave greater reduction of hydraulically reversible and irreversible fouling resistance than the standalone treatments, and MIEX followed by BAC gave greater reduction of both fouling resistances. With successive filtration cycles, the reversible and irreversible fouling resistance increased for all treatments, with high resistance being correlated with low flux.

#### 3.2.3. Determination of Unified Membrane Fouling Index

**Figure 3:**
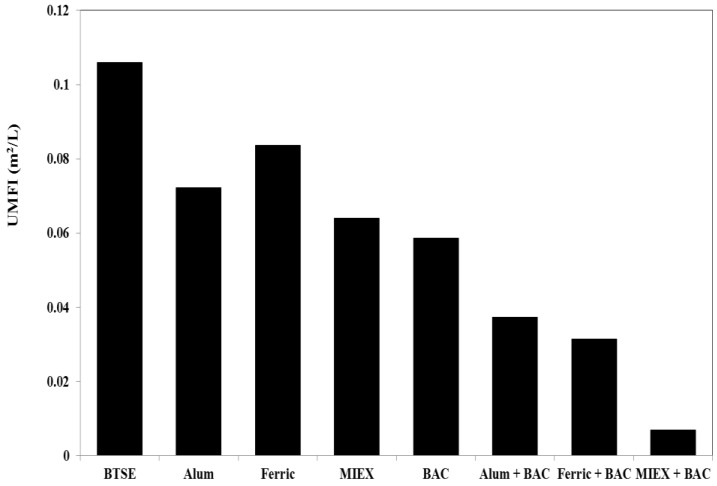
Unified membrane fouling index (UMFI) values for total fouling.

The unified membrane fouling index (UMFI) values for total fouling for the different treatments were determined after filtration cycle 1. Using the relationship developed by Huang et al. [31], whereby the UMFI can be calculated from the linear fitting of *J_o_*/*J* versus specific volume (V), the values for after the various treatments were determined. A higher UMFI value generally indicates greater total membrane fouling. Ferric chloride gave less reduction in UMFI than the alum treatment, which gave less than the MIEX treatment (Figure 3). BAC led to 42% reduction in UMFI, i.e., slightly greater than MIEX treatment, which, in turn, was greater than for coagulation.

Consistent with the flux and filtration resistance, there was a marked reduction in total fouling for the sequential treatment processes. The decrease in UMFI was 65%, 70% and 93% for alum, ferric chloride and MIEX, respectively, when combined with BAC treatment, clearly demonstrating that MIEX pre-treatment led to greater reduction in UMFI than coagulation followed by BAC treatment.

In order to better understand the impact of pre-treatment on fouling reduction, the proportion of fouling per mg DOC remaining in the feed was calculated. The UMFI value per mg DOC was 0.0091 m^2^/mg for BTSE. The UMFI value per mg DOC reduced to 0.0074, 0.0088, 0.0089 and 0.0068 m^2^/mg after alum, ferric chloride, MIEX and BAC treatment, respectively. Hence, the value was decreased after pre-treatment of the feedwater, and BAC was very effective for reducing membrane foulants. As shown in Section 3.2.1, the trend for the improvement of the flux for cycle 1 was BAC > MIEX > alum > ferric whereas for the UMFI value per mg DOC it was BAC > alum > ferric = MIEX. A similar finding was reported in our earlier study that BAC gave lower removal of DOC (31%) than granular activated carbon (74%), but it led to significantly greater improvement in flux [16]. This provides further evidence that the characteristics of the organics in the feedwater play a more significant role in the fouling of membrane than the DOC concentration alone. Similar to the standalone processes, the sequence of coagulation or MIEX followed by BAC further reduced the UMFI value per mg DOC, and MIEX with BAC treatment gave a markedly lower value than coagulation with BAC treatment.

### 3.3. Characterisation of Organics Using Advanced Techniques

#### 3.3.1. ATR-FTIR

**Figure 4:**
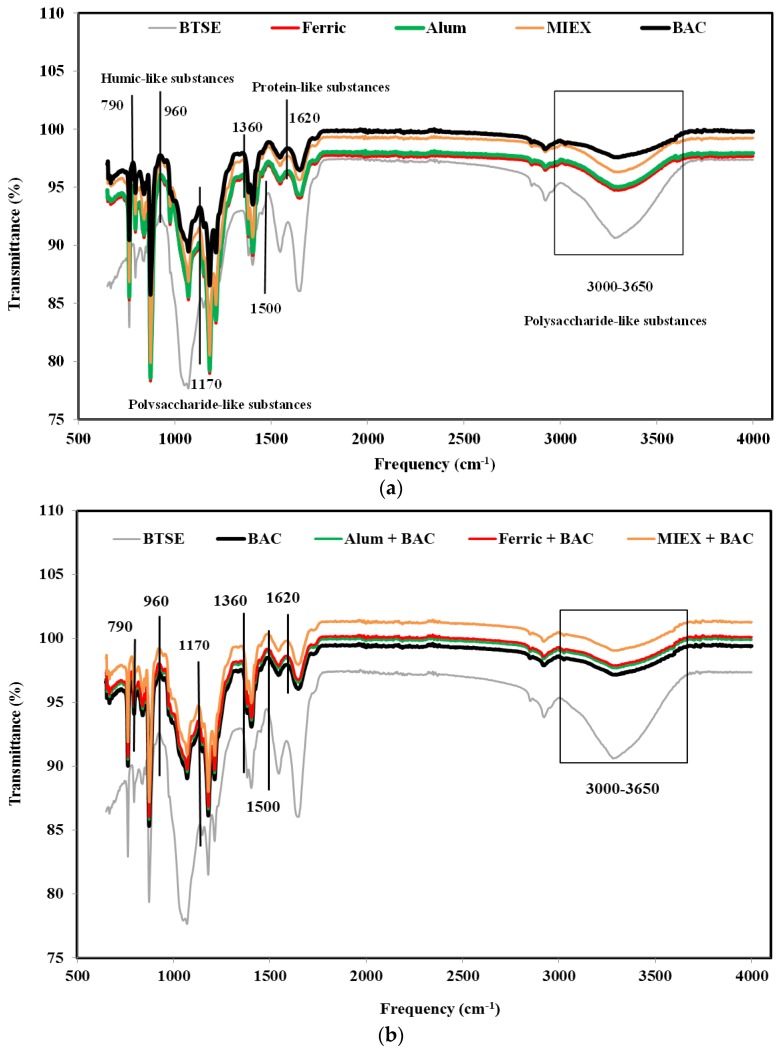
FTIR spectra of fouled MF membranes after (**a**) standalone treatments and (**b**) the sequential treatments.

ATR-FTIR spectroscopy was used to identify the major foulant groups retained reversibly and irreversibly on the membrane surface after the various pre-treatments. The contribution of the foulants was determined by subtracting the spectrum of a pristine membrane from that of the fouled membrane after cycle 1. Using the assignation of spectral bands according to Cho et al. [32] and Howe et al. [33], it was shown that functional groups associated with polysaccharide-like substances, protein-like substances and humic substances were present on the membranes. BAC treatment increased the transmittance of the assigned peaks more than MIEX treatment or coagulation with alum or ferric chloride (particularly for polysaccharide-like substances at 3000–3650/cm), indicating that there were less of these substances residing in the foulant layer (Figure 4a). This was also confirmed by the mass balance result which showed that the rejection of the organics (in terms of DOC, protein and carbohydrate) was greater for coagulation than for MIEX, which, in turn, was greater than for BAC (Figure S1).

The transmittance at the assigned bands was significantly lower for the combinations of coagulation or MIEX with BAC treatment than the standalone processes (Figure 4b), indicating that the sequential treatment process was more effective for removing the organic matter resulting in less deposition of organics on the surface of the MF membrane. MIEX followed by BAC treatment led to lower rejection of these organics compared with the BAC followed by coagulation treatment, consistent with the lower degree of fouling.

#### 3.3.2. Size Exclusion Chromatography

**Figure 5:**
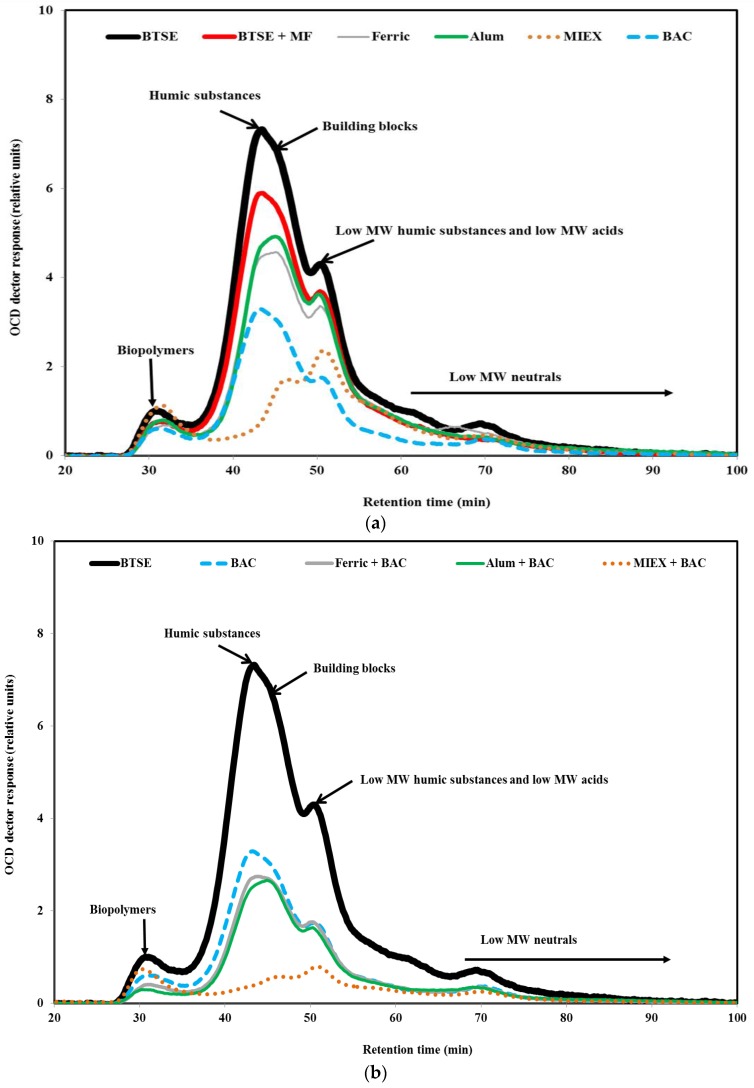
LC-OCD chromatograms of (**a**) BTSE, BTSE MF permeate and BTSE after the various standalone treatments; and (**b**) BTSE after the sequential treatments.

LC-OCD analysis was performed in order to understand the effect of pre-treatment on the molecular weight distribution of the organic matter. As seen in Figure 5, a greater proportion of the biopolymers (protein-like material, amino sugars, polysaccharide-like material, ≥20,000 Da) than humic substances (1000–20,000 Da) was retained by the membrane, followed by building blocks (breakdown materials of humic substances, 350–500 Da) and low MW organics (acids and neutrals, <350 Da), indicating that these high MW biopolymers and humic substances played a vital role in the fouling of MF membrane. These high MW organics lead to reversible fouling as they tend to form a gel layer on the membrane surface, whereas low MW organics would have blocked the membrane pores partially or completely which contributed to irreversible fouling resistance. Moreover, some smaller molecules may be prevented from entering the membrane structure by the layer of biopolymers [34]. The contributions to the total DOC for the BTSE were 9% by biopolymers, 43% by humic substances, 12% by building blocks and 36% by low MW organics.

To obtain a better understanding of the composition of the organic matter after the pre-treatments, the relative proportions of each fraction of the organics were determined to reveal their role in fouling the membrane. After coagulation, the ratios of biopolymers/DOC and humics/DOC were 0.100 and 0.522 for alum, and 0.098 and 0.512 for ferric chloride, respectively, indicating that the ferric chloride-treated effluent had very slightly lower proportions of humic substances. The ratios of biopolymers/DOC and humics/DOC were 0.187 and 0.224, respectively, after MIEX treatment. This indicates that it was highly effective for removing humic substances. After BAC treatment, the ratios of biopolymers/DOC and humics/DOC were 0.095 and 0.522, respectively, and indicate that the BAC effluent had a slightly lower proportion of biopolymers compared with coagulation. As mentioned in Section 3.1, the ratios of protein and carbohydrate to DOC were lower for BAC than coagulation, which, in turn, were lower than for MIEX. It may be concluded that BAC treatment led to lower fouling per mg DOC (Section 3.2.3) due to preferential removal of the biopolymers.

In terms of absolute concentration, BAC treatment led to greater removal of biopolymers than coagulation, and alum and ferric chloride had approximately similar removal efficiency for biopolymers (Figure 5a). On the other hand, MIEX treatment did not remove biopolymers, probably because the large molecules are unable to penetrate into the pores of the resin, and their neutral and/or hydrophilic character [28]. It was noted that MIEX could reduce the concentration of the hydrophilic organics (Figure S2 Organic fractions in BTSE and variously treated-BTSE samples). This was probably due to the low MW of some protein and carbohydrate molecules, which enabled them to penetrate into the pores of the resin. The removal efficiency of humics and building blocks was greater for MIEX than for BAC or coagulation. Consistent with this resin fractionation showed that removal of the HPO fraction was substantially better for MIEX than BAC or coagulation (Figure S2). The fluorescence spectra also provide evidence that MIEX led to significantly greater removal of fluorescent humic acid-like and fulvic acid-like substances than the BAC treatment or coagulation (Figure S3 EEM spectrum volumes of the untreated and variously treated BTSE samples). MIEX gave less removal of building blocks than humic substances. This is because building blocks contain a higher proportion of amine functionalised compounds than the humic fractions, and the anionic exchange character of the MIEX resin would adsorb organic acids in preference to amine functional groups [35]. The removal efficiency of low MW organics was significantly higher for BAC than for both coagulants. MIEX was ineffective for reducing the low MW neutral organics due to their neutral charge. When the coagulated or MIEX-treated sample was subjected to BAC treatment, there was further removal of biopolymers, humics and building blocks (Figure 5b).

## 4. Conclusions

All pre-treatments (coagulation, MIEX and BAC) led to reduction in the reversible and irreversible fouling of the MF membrane by the secondary effluent. MIEX treatment gave greater reduction of fouling resistances than coagulation, due to the greater removal of humic substances. However, greater improvement in flux was obtained with BAC treatment than with coagulation or MIEX treatment. This was attributed to the breakdown of biopolymers by micro-organisms and adsorption of those molecules on the biofilm, and adsorption of humic substances by activated carbon, hence lower amounts of these foulants deposited on the membrane surface. BAC effectively removed low MW organics, which may decrease the risk of biofouling in subsequent processes or use of the water, whereas coagulation and MIEX were less effective for removing these organics.

Sequential coagulation or MIEX and BAC treatment provided further marked reductions in reversible and irreversible membrane fouling resistance. The addition of MIEX prior to BAC treatment gave greater removal of DOC, protein and carbohydrate, thus contributing greater flux improvement, which would reduce the frequency of hydraulic or chemical cleaning of the membrane and thus help in extending the membrane lifetime. This work was based on a single dose of MIEX and a single EBCT for BAC, both of which were higher than typically used; therefore, further work would be required to determine the optimum conditions and sequence of the two unit processes.

## Figures and Tables

**Table 1:** Characteristics of the BTSE sample before and after various treatments.

Treatment	DOC (mg/L)	UVA_254_ (/cm)	SUVA (L/m·mg)	Colour (Pt-Co Units)	Protein (mg·BSA/L)	Carbohydrate (mg·glucose/L)
BTSE	11.79 ± 0.12	0.271 ± 0.003	2.29	86 ± 2	14.63 ± 0.31	12.25 ± 0.41
BAC	8.64 ± 0.08	0.169 ± 0.005	1.95	29 ± 2	9.22 ± 0.20	8.33 ± 0.25
Alum	9.87 ± 0.09	0.216 ± 0.006	2.19	41 ± 1	11.45 ± 0.25	9.80 ± 0.32
Ferric chloride	9.61 ± 0.11	0.208 ± 0.004	2.16	37 ± 2	10.95 ± 0.25	10.41 ± 0.31
MIEX	7.28 ± 0.12	0.089 ± 0.007	1.22	13 ± 1	9.72 ± 0.16	9.02 ± 0.34
Alum + BAC	7.15 ± 0.07	0.120 ± 0.010	1.68	16 ± 1	6.73 ± 0.25	6.42 ± 0.30
Ferric chloride + BAC	7.59 ± 0.07	0.114 ± 0.010	1.50	10 ± 1	6.52 ± 0.30	7.17 ± 0.39
MIEX + BAC	6.01 ± 0.07	0.063 ± 0.001	1.04	6 ± 1	5.42 ± 0.20	6.12 ± 0.24

Note: BAC = biological activated carbon; BTSE = biologically treated secondary effluent; SUVA = specific ultraviolet absorbance.

## Supplementary Materials

The following are available online at www.mdpi.com/2077-0375/6/3/39/s1, Table S1: Properties of the activated carbon used in the column, Table S2: DOC removal using alum and ferric chloride coagulation, Figure S1: Distribution of organics in reversible and irreversible fouling after MF (a) DOC (b) protein and (c) carbohydrate contents (data points are average values of duplicate samples), Figure S2: Organic fractions in BTSE and variously treated-BTSE samples (data points are average values of duplicate samples), Figure S3: EEM spectrum volumes of the untreated and variously treated BTSE samples.
