# Supplementary material for: Combining Coagulation/MIEX with Biological Activated Carbon Treatment to Control Organic Fouling in the Microfiltration of Secondary Effluent
Source: Membranes (Basel). 2016 Jul 30;6(3):39. doi: 10.3390/membranes6030039 (PMC5041030; doi:10.3390/membranes6030039)
Supplement: Supplementary file 1 [file membranes-06-00039-s001.pdf]

Supplementary Materials: Combining Coagulation/MIEX with Biological Activated Carbon Treatment to Control Organic Fouling in the Microfiltration of Secondary Effluent

Biplob Kumar Pramanik, Felicity A. Roddick and Linhua Fan

Table S1. Properties of the activated carbon used in the column.

Physical Properties	Virgin GAC
Surface area (BET m^2/g)	1017
Total pore volume (cm^3/g)	0.716
Micropore volume (cm^3/g)	0.297
Micropore content (%)	93
Mesopore content (%)	5
Macropore content (%)	2

Table S2. DOC removal using alum and ferric chloride coagulation.

Coagulation	pH	Dosages (mg/L)			
		2.5 mg/L	5 mg/L	7.5 mg/L	10 mg/L
Alum	6	10.4	14.5	16.3	17.4
	5	12.5	16.3	17.8	18.4
	4	12.6	16.1	17.2	17.8
Ferric Chloride	6	14.6	15.8	18.1	19.8
	5	14.4	18.5	19.7	21.2
	4	13.6	19.2	20.1	21.8

Mass balance for organics in the filtration system

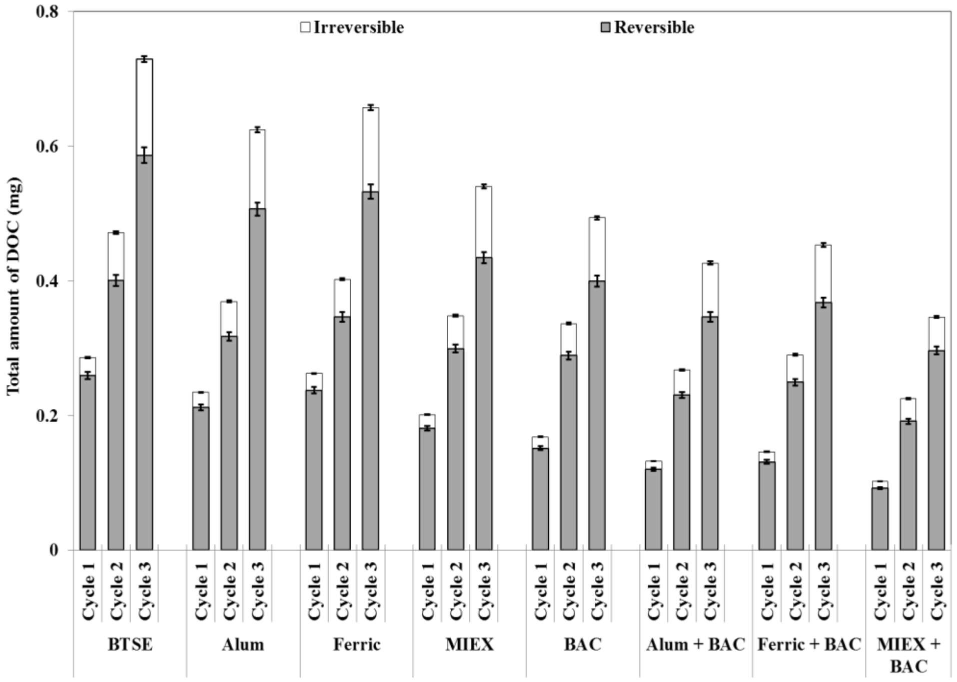

(a)

Figure S1. *Cont.*

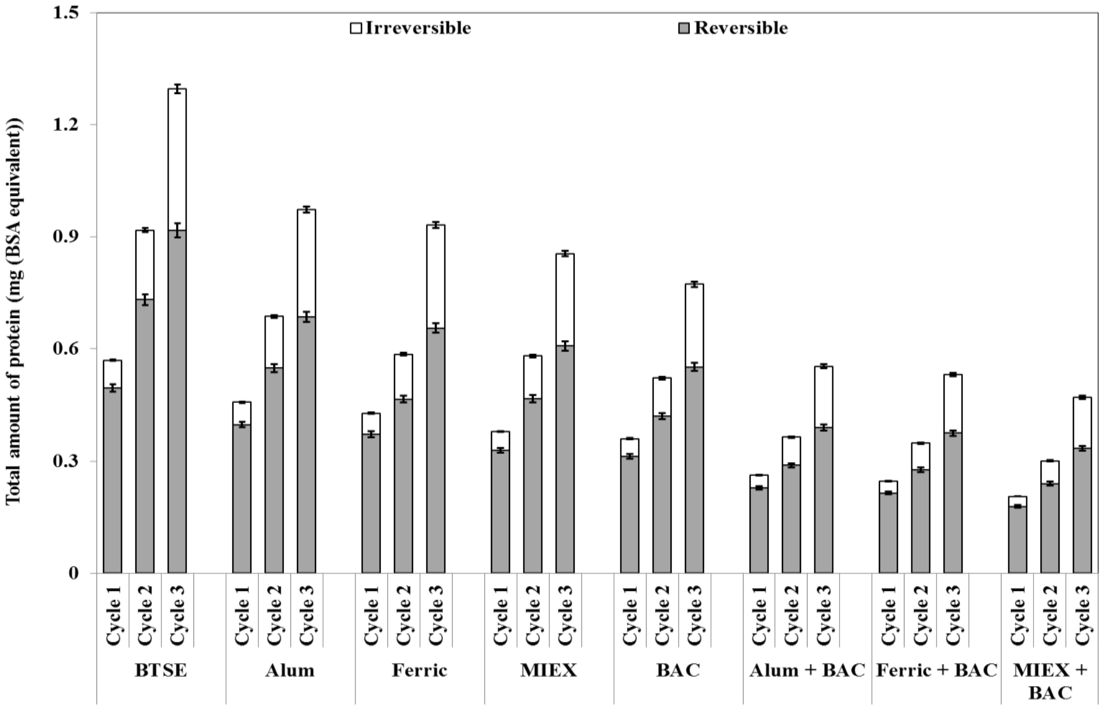

(**b**)

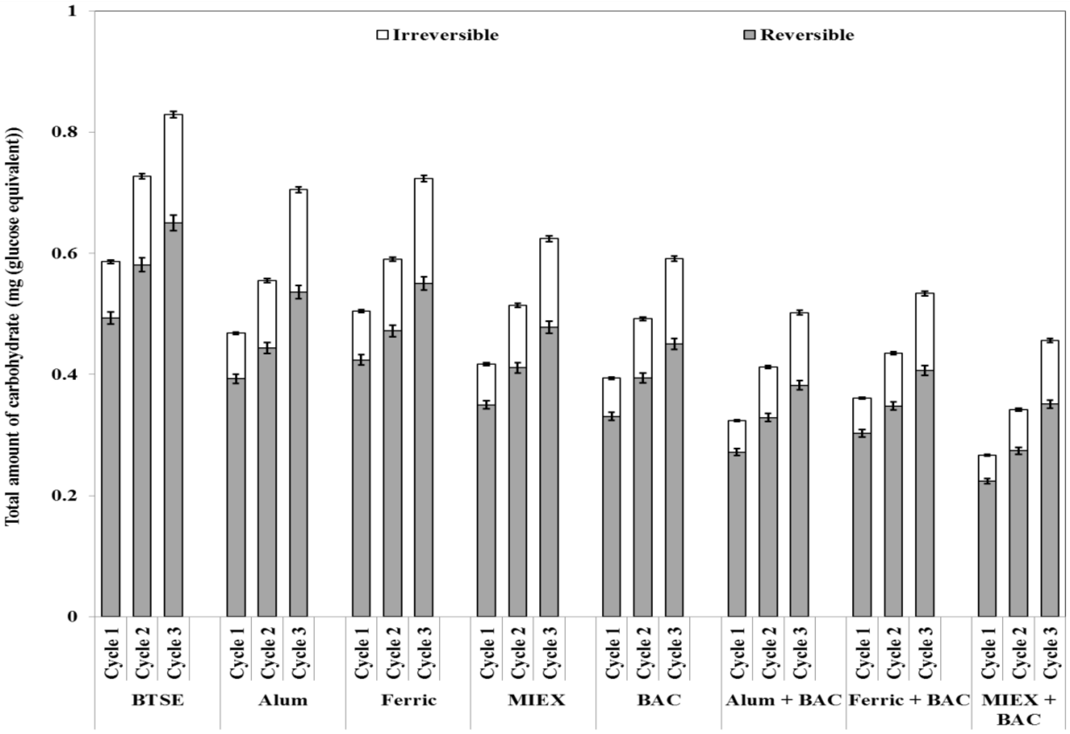

(**c**)

Figure S1. Distribution of organics in reversible and irreversible fouling after MF (**a**) DOC; (**b**) protein and (**c**) carbohydrate contents (data points are average values of duplicate samples).

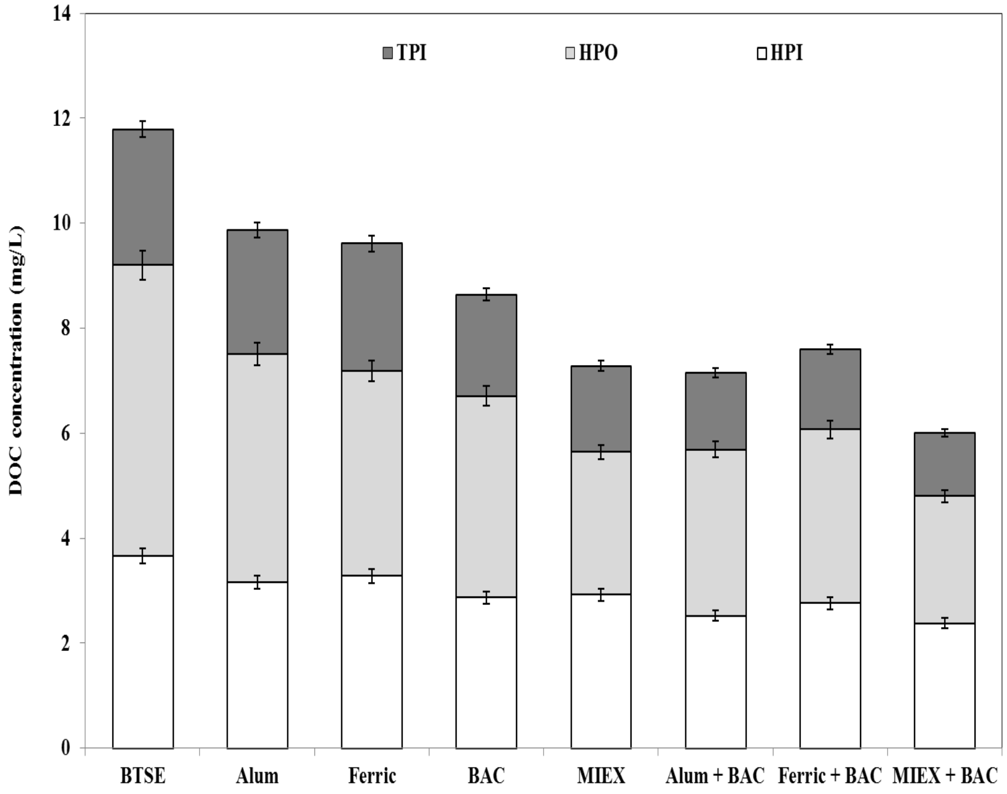

Figure S2. Organic fractions in BTSE and variously treated-BTSE samples (data points are average values of duplicate samples) (Note: HPI = hydrophilic, HPO = hydrophobic, TPI = transphilic).

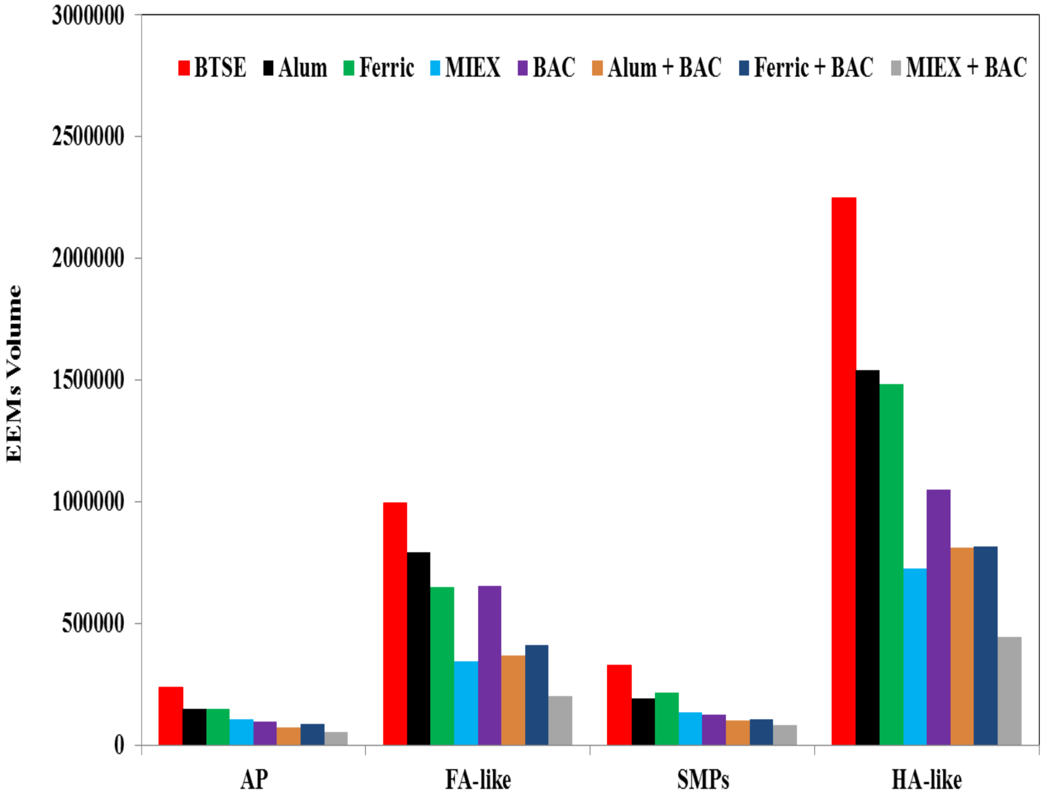

Figure S3. EEM spectrum volumes of the untreated and variously treated BTSE samples. (Note: AP = aromatic protein, FA = fulvic acid-like, SMPs = soluble microbial products, HA = humic acid-like).
